# Proximal femoral focal deficiency – a rare congenital entity: two case reports and a review of the literature

**DOI:** 10.1186/s13256-020-2350-y

**Published:** 2020-02-05

**Authors:** Felix U. Uduma, Edwin M. Dim, Ngozi R. Njeze

**Affiliations:** 1 Department of Radiology, Faculty of Clinical Sciences, College of Health Sciences, University of Uyo, Uyo, Nigeria; 2 Department of Trauma and Orthopaedics, Faculty of Clinical Sciences, College of Health Sciences, University of Uyo, Uyo, Nigeria; 3 Department of Radiation Medicine, Faculty of Medical Sciences, College of Medicine, University of Nigeria, Enugu Campus, Enugu, Nigeria

**Keywords:** Proximal femoral focal deficiency, Femur, Radiographs

## Abstract

**Background:**

Proximal femoral focal deficiency is an extremely rare congenital anomaly with an incidence of 1.1–2.0 in 100,000 live births. It is a dysplastic phenomenon with predilections for the proximal two-thirds of the femur leading to limb length discrepancies.

We report two cases of proximal femoral focal deficiency, which is a rare entity.

**Case presentations:**

**Case 1**

A 4.5-month-old baby Annang tribe girl was referred in April 2019 to our Radiology Department, University of Uyo Teaching Hospital, Nigeria for lower limb radiographs. This was on account of her shortened left lower limb from birth despite uneventful antenatal history. An examination revealed bulky left thigh with abduction of her left hip joint.

Radiographic evaluations showed absent left femoral capital epiphysis, with deficient proximal left femur. A diagnosis of proximal femoral focal deficiency was made. Sadly, the parents and baby failed to honor future orthopedic consultations on intimation of sequential management protocols.

**Case 2**

A 4-month-old baby Ibibio tribe girl was similarly referred in August 2019 to the same Radiology Department for lower limb conventional radiographs due to short left lower limb that was noticed from birth. An examination showed shortened left lower limb in external rotation. Her right and left lower limbs measured 27 cm and 23 cm, respectively, with landmark taken from anterior superior iliac spine to tip of medial malleolus. A diagnosis of proximal femoral focal deficiency was made. Corroborating radiographs showed shortened and hypoplastic left femoral shaft but preserved femoral capital epiphysis. Coincidentally, the parents have not brought back their baby to our orthopedic clinic.

**Conclusions:**

The discovery of two cases of proximal femoral focal deficiency, a rare entity, from referrals for conventional radiography in our Radiology Department encourages literature documentation. Such recognition will facilitate early institution of management, thus ensuring meaningful childhood growth.

## Background

Proximal femoral focal deficiency (PFFD) is a subset of a broader group called congenital femoral deficiency. PFFD is also known as congenital proximal femoral deficiency (CPFD) [1]. It is a rare congenital anomaly with an incidence of 1.1–2.0 in 100,000 live births [2–5]. PFFD has a female bias with a male-to-female ratio of 1:2 [6]. Most cases of PFFD are unilateral (85–90%); PFFD is rarely bilateral [2, 7]. When unilateral, the right femur is the most frequent culprit [6].

PFFD is a dysplastic phenomenon with a spectrum of femoral involvement. This ranges from hypoplastic shortened femur to total absence of proximal two-thirds of femur. Severe affectation may even culminate in femoral agenesis [6]. Patho-anatomically, it hinges on defective primary ossification center-cartilage anlage [5]. The consequence of this dysplasia is impaired childhood growth, abnormal gait, cosmetic implications and psychosocial behavioral changes.

Although a rare and sporadic case, it is intriguing, but serendipitous, on our part to have two cases of PFFD in our hospital in less than 6 months. This, therefore, necessitates adding to the existing literatures on PFFD, especially with a dearth of such reports in West African sub-region [6].

We aim to report two cases of unilateral focal femoral shortening identified between April and August, 2019 at the University of Uyo Teaching Hospital (UUTH), Uyo, Nigeria.

## Case presentations

### Case 1

A 4.5-month-old baby Annang tribe girl was referred by our Orthopedic Unit, UUTH, Uyo to the Radiology Department of the same hospital in April 2019. This referral was for radiographic evaluations of her lower limbs with inclusion of the pelvis. The main clinical presentation was a history of shortened left lower limb from birth with gradual progression of deformity. She was a full-term spontaneous vertex delivery of a non-consanguineous marriage. There was no antenatal history of maternal diabetes, non-prescribed drug intake, or perinatal exposure to radiation. There was no history of trauma to or fall of her mother during the antenatal period. There was no positive family history of previous similar occurrence. Her four older siblings do not have any shortened limbs.

**Fig. 1:**
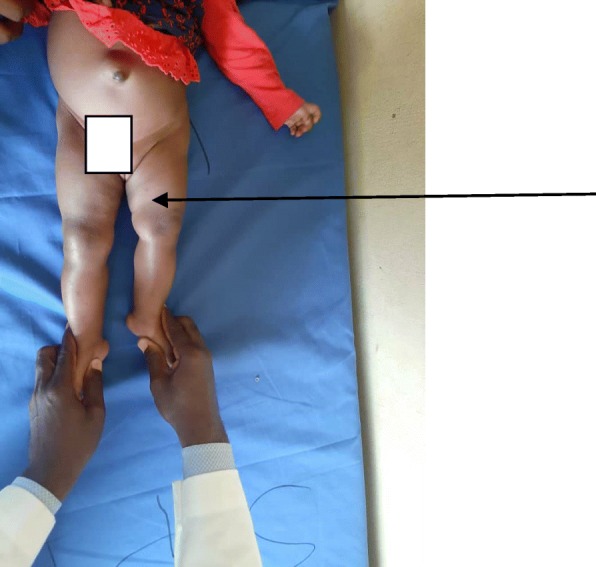
Clinical photograph of Case 1 reveals shortened left lower limb (*arrow*). The left thigh is bulky with flexion, lateral rotation and abduction at the left hip joint

On physical examination, her left thigh was bulky with flexion, lateral rotation, and abduction of the left hip joint (see Fig. 1). The range of movements at her left hip was limited fixed flexion deformity but no limitation of left knee joint flexion. Her left ankle showed limitation of ankle dorsiflexion. Both upper limbs show no anomaly with normal limb length and normal range of movements. She has normal head size with no craniosynostosis.

**Fig. 2:**
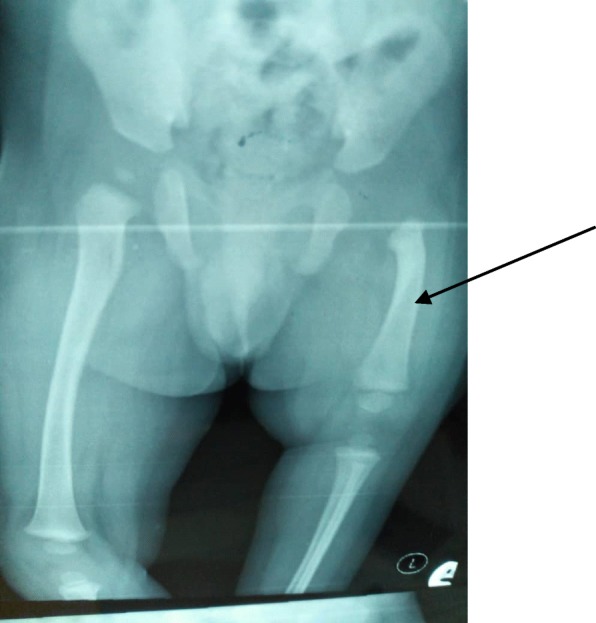
Anterior posterior radiograph of the pelvis and thighs showing short left femur with deficiency of its proximal two-thirds (*arrow*) and bowing of residual shaft. Note non-visibility of left femoral capital epiphysis as well as attendant shallow left acetabulum

Radiological evaluations showed deficient proximal left femur with lateral bowing of its residual shaft. The left femoral capital epiphysis could not be visualized vis-à-vis the contralateral side (see Fig. 2). On reporting to our Orthopedic clinic with the radiographs, the management protocol, which is a multiple step procedure, was explained to the parents to which they acquiesced. Sadly, the parents and the infant have not visited our Orthopedic clinic again. In early December 2019, the managing team successfully located the house of the parents with the intention of encouraging them to revisit the clinic. They confessed patronizing a traditional bone setter and rebuffed such invitations. The infant was not in the house, which would have afforded the authors the benefit of acquiring interval clinical photographs.

### Case 2

A 4-month-old baby Ibibio tribe girl was referred in August 2019 to our Radiology Department, UUTH, Uyo, Nigeria from the Orthopedic Unit for plain radiography of her lower limbs; this was on account of her short left lower limb noticed from birth. Her parents said that her left lower limb was getting progressively shorter but she had no other abnormalities. Antenatal history, past medical history, and family history were not remarkable. There was no history of trauma to the mother during pregnancy or perinatal exposure to radiation. Developmental milestones had been normal so far.

**Fig. 3:**
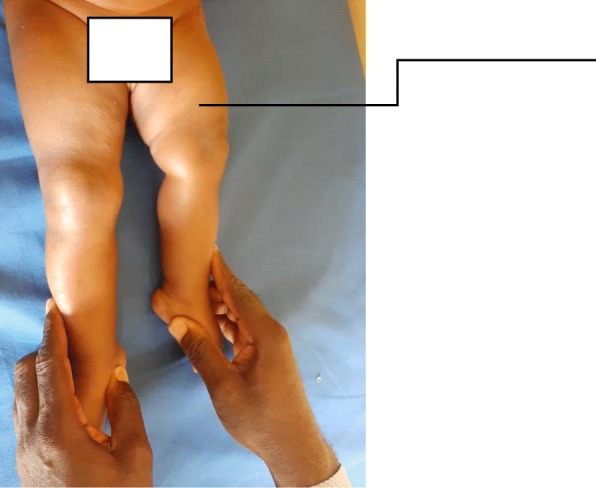
Clinical photograph of a 4-month-old baby girl with shortened left lower limb and external rotation/abduction of the left thigh (*arrow*)

On physical examination, she appeared otherwise normal and well nourished. Both her lower limbs were actively mobile. Her left lower limb was shortened and in external rotation (see Fig. 3). Her right lower limb measured 27 cm from the anterior superior iliac spine to the tip of medial malleolus, whereas her left lower limb measured 23 cm on similar landmarks. Normal range of motion of both knee joints and hip joints were observed. Hip abduction was full and back examination was normal. Her upper limbs showed no anomaly with full range of movement. Aside from umbilical hernia, no other congenital abnormalities were seen on abdominopelvic ultrasonography. A diagnosis of PFFD was made and she was referred for radiographic examinations.

**Fig. 4:**
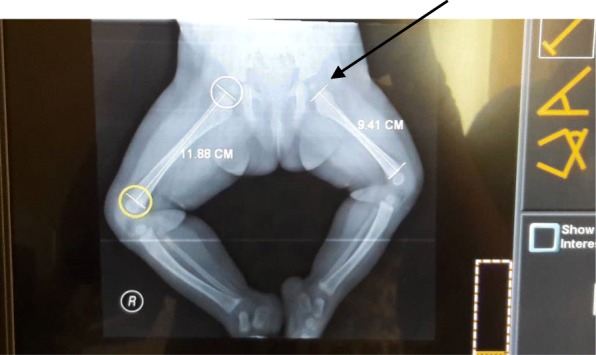
Frog-leg lateral radiograph of the pelvis and lower limbs of Case 2 showing a normal but short left femur with shortening of 2.5 cm. Note that the acetabulum and femoral capital epiphysis were well developed bilaterally (*arrow*)

Plain radiographs of her thigh and lower limbs showed shortened and hypoplastic left femur with preserved left femoral capital epiphysis. Her left femur had normal configuration but had a shorter shaft than the contralateral femur. The shaft of her left femur measured 9.41 cm whereas the shaft of her right femur measured 11.88 cm (see Fig. 4).

On receipt of the radiographs, the possible plans of management were discussed with her parents. Unfortunately, they have not kept any further appointments. Recent telephone communications in December 2019 with the parents revealed that the child has been left to her fate. This is because of the parents’ belief of spontaneous limb lengthening.

## Discussion

PFFD, as mentioned earlier, is an extremely rare congenital anomaly with few case reports in the literature [2]. Therefore, for us to have two cases in a short period of less than half a year merits documentation and inclusion in existing literatures. Interestingly, our two patients showed various degrees of involvement, as is known in PFFD [4]. Our first index patient showed severe deficiency of proximal two-thirds of left femur (see Figs. 1 and 2) whereas the second case only showed mild shortening of the left femur (see Figs. 3 and 4). PFFD features are recognized to vary from mild shortening to severe deficiency of the femoral head, acetabulum, and femoral shaft [7, 8].

The diagnosis and classification of PFFD have been majorly based on conventional radiographic features [9]. As such, there are different classifications from different authors. However, the commonly applied classification is Aitken’s classification A to D [1, 4, 5, 10, 11]: PFFD class A – presence of femoral head with varus deformity; class B – presence of femoral head but with delayed ossification; varus, mild acetabular dysplasia, and pseudarthrosis may occur; class C – absence of femoral head as well as acetabular dysplasia and shortening of femur; and class D – absence of femoral head with severe dysplastic and severely shortened femur [5, 10]. This therefore means our indexed patient 1 belongs to Aitken’s class C while indexed patient 2 belongs to class A. The variance in spectra is anchored pathogenetically on failure of formation. PFFD is a failure of formation that is based on sclerotome subtraction theory (early damage of the neural crests of L4, L5) with defective chondrocyte proliferation and maturation in the proximal femoral growth plate [2].

There are known risk factors for PFFD. Some of the inciting teratogenic agents for PFFD are hypoxia, ischemia, diabetes mellitus, irradiation, microbiological agents, chemical toxicities, hormones, mechanical injuries, thermal injuries, thalidomide exposure, and trauma to the fetus between the fourth and eighth weeks of gestation [2, 3]. Interestingly, none of these agents could be linked to either of our index patients or their mothers. PFFD is not a chromosomal abnormality but may be associated with other congenital abnormalities or syndromes [2]. The most frequent associated abnormality is fibular hemimelia: Online Mendelian Inheritance in Man (OMIM) 228900. This is because the fibular field of the limb bud controls the development of the proximal femur [12]. Other syndromes and associations of PFFD are femoral–fibula–ulna syndrome (OMIM 228200), femoral–facial syndrome (OMIM 134780), coxa vara (OMIM 122750), oligodactyly (OMIM 176240), absent patella (OMIM 161200), absence of cruciate ligaments, spinal deformities, congenital knee instability, club foot deformities (OMIM 119800), and limb/pelvis-hypoplasia/aplasia syndrome (OMIM 276820) [2–5, 13]. PFFD has also been seen to have co-existed with Mayer–Rokitansky–Küster–Hauser (MRKH) syndrome (OMIM 277000) [14]. This is a Müllerian duct anomaly whereby the uterus, fallopian tube, cervix, and vagina are congenitally absent.

On clinical examination, PFFD shows as limb discrepancies with short bulky affected thigh. Significant variability in the clinical presentations is based on the degree of femoral deficiency [15]. The lower extremity may be flexed, abducted, and externally rotated [5, 7]. Instability of the hip and knee joints may be seen. Mental disorders are not usual characteristics [3].

The diagnosis of PFFD requires the presence of a shortened femur with proximal femoral deficiency. Deficiency of iliofemoral articulation, leg length discrepancy, limb malrotation, and varus deformity at the sub-trochanteric level may also be seen [7]. These features are truly shown on radiographic assessment, thus permitting definite classification even during the first year of life, as shown in our index patients (Figs. 2 and 4). The most commonly used classification scheme is that of Aitken. This scheme is based upon the presence and location of femoral head and neck on conventional radiography and provides an assessment of future limb function. It is also useful in planning treatment. However, Aitken’s classification scheme relies on conventional radiography, which makes it cumbersome to classify a child prior to skeletal maturity which is often delayed in children with PFFD [16]. This is significant because the earlier the detection and treatment of PFFD, the earlier normal growth can start [16]. This, therefore, calls for complementary investigations like magnetic resonance imaging (MRI), MRI arthrography, three-dimensional volumetric computed tomography (CT), arthrography, and ultrasonography. These permit a more complete characterization of the anatomic abnormalities in these skeletally immature patients.

The multi-planarity and non-ionizing characteristics of MRI make it the more definitive and preferred modality for evaluation of PFFD. Usually, coronal and axial sections suffice [17, 18]. MRI examination evaluates the size and configuration of the cartilaginous anlage of the acetabulum and femoral head as well as the congruency of the femoroacetabular joint [7]. MRI is used to define the cartilaginous proximal femur and the presence or absence of a cartilaginous connection to the femoral head, thus guiding therapeutic decisions [5]. MRI shows variable signal intensity depending on the fat and collagen compositions of the cartilaginous connections. Gradient echo (GRE) MRI clearly depicts cartilages [5]. Characteristic patterns of adjacent soft tissue abnormalities are succinctly shown on MRI. In PFFD, all muscles will be present but most will be smaller than their normal counterparts; however, exceptions are hypertrophied sartorius and elongated obturator externus muscle, which will remain muscular almost up to its insertion [19]. Magnetic resonance (MR) arthrography is useful for confirming the presence or absence of a femoral head and may be used as an adjunct to standard non-contrast MRI [5]. A conventional arthrogram shows the cartilaginous portion in PFFD but it is invasive. CT is only useful at an older age when the acetabulum and proximal femur are nearly fully ossified. Three-dimensional CT reconstruction is useful to compare the normal acetabulum with the dysplastic side. Ultrasonography, on the other hand, has the capability of prenatal diagnosis of PFFD. This is easier after the second trimester of pregnancy [3, 20, 21].

PFFD poses a significant challenge to effective treatment [6]. It demands a multidisciplinary approach of prosthetists, pediatric orthopedic surgeons, and physical therapists. The guiding factors in PFFD management depend on the degree of femoral shortening, musculature, foot deformities, and the status of the hip and knee joints [22, 23]. Therefore, management hallmarks will be equalizing limb length, correcting rotational anomalies, pelvofemoral stability, and stabilizing the feet [5]. The whole essence is to achieve improved functional ambulation with least energy expenditure. This may translate to an individualized primary treatment. However, the core mandate is the early institution of therapy so as to achieve adequate growth of the femur [8].

Since the most apparent functional deficit in PFFD is shortened limb, as mentioned previously, this creates room for non-surgical treatment. Thus the first step in most treatments of a child with PFFD coincides with the time when the child is ready to stand [24]. The child will be fitted with an extension prosthesis to equalize leg lengths and allow standing and walking [24]. This is done irrespective of any intended future management as the initial treatment should synchronize with normal development. This acts as a stop gap while waiting for the child to reach an appropriate age for surgery or between episodes of surgical treatment [25].

Broadly, surgical management of PFFD could be limb lengthening or limb modification. Limb lengthening is a complex reconstruction of the hip and knee joints with limb lengthened up to 25 to 30 cm [22], whereas limb modification involves foot amputation, knee arthrodeses, limb rotationplasty, hip reconstruction, and iliofemoral arthrodesis [4, 15, 22]. For mild cases, reconstruction of the hip with femoral or pelvic osteotomy is possible [22].

Limb lengthening with or without contralateral epiphysiodesis will be appropriate for a child if the predicted limb discrepancy at maturity does not exceed 20 cm, and the hip is stable with relatively good knee, ankle, and foot [26]. Otherwise, preparatory surgery of the hip and knee are required before initiating lengthening. Examples of these preparatory surgeries are pelvic osteotomy for acetabular dysplasia and correction of hip anomalies like retroversion, flexion contraction, and abduction contraction [26]. Before lengthening, the proximal femur should be ossified as normal for that age since there is usually delayed femoral neck and subtrochanteric ossification in PFFD [26]. Therefore, preparatory surgeries are usually performed between 2 and 3 years of life [26]. Lengthening is done a year after the preparatory surgery except if the femur is excessively short or the femoral neck has not ossified, then there will be a wait of a year or 2 years [26]. Lengthening should be multiple and serial. It is recommended that lengthening should be limited to a maximum of 5–8 cm during one treatment, or 20% of the original length of the femur, to reduce the risk of complications [26]. The required number of lengthenings which must be completed at high school demands prediction of length discrepancy at maturity [26]. For ease of parents’ remembrance of child age for lengthening, a rule of thumb (a rule of 4 years) is adhered to with lengthening done every 4 years [26]; this means the second and third lengthenings will be done at 8 years and 12 years of life, respectively. Since each lengthening achieves 8 cm, then the gain of three lengthenings will be 24 cm at 12 years [26]. One centimeter gain from preparatory hip surgery will add up to 25 cm [26]. If limb length discrepancy is greater than 25 cm, then physiodesis of the contralateral distal femoral growth plate will give an additional gain of 5 cm adding up to 30 cm [26]. Any further deficit will mandate one more lengthening that will be at 16 years of age according to the rule of thumb.

Limb modification surgery for PFFD includes Van Nes rotationplasty. This rotationplasty or Van Nes’ so-called turn-about procedure is done for patients with severe deformity with the aim of levelling the ankle of the involved side with the knee of the uninvolved side [25]. It is a conversion of the knee to a hip joint by flexing it 90 degrees and fusing the femur to the pelvis and conversion of the foot into the knee joint, followed by fitting the short limb with a leg prosthesis [2, 22]. Contraindication to rotationplasty is bilateral PFFD [27]. However, the shortcomings of Van Nes rotationplasty are inability to achieve sufficient rotation during surgery and possible de-rotation with continued growth [27]. In such shortcomings and failed Van Nes procedure, Syme amputation serves as the salvage procedure [26]. Van Nes rotationplasty procedures allow the use of a below-the-knee prosthesis with a biologic knee [24], whereas above-the-knee prosthesis with a mechanical knee is achieved with a combination of Syme or Boyd’s amputation, knee fusion, and possibly an epiphysiodesis at the knee [26]. It is noteworthy that surgical reconstruction is known to result in a good prognosis [3, 7, 22].

The strength of this study revolves around the aforementioned rarity of this condition. The treatment of these cases at early ages would have been of immense contribution to good management and favorable prognosis.

### Limitations

The limitation of this study is the fact that the parents absconded with the babies. Although it is their prerogative to seek treatment in any desired place, we lost the privilege of managing these patients, assessing management outcome, and evaluations of childhood growth. We are compelled to rationalize that the parents must have resorted to traditional bone setters. This is a commonplace festering problem in our local clime. They only resort to orthodox bone treatment when faced with complications. We have resorted to advocacy to stem the tide. Another limitation is that radiographic evaluation tends to overestimate the degree of deficiency [17]. The apparent loss of continuity between the femoral shaft and the head/neck is the fibrocartilaginous tissue which is unrecognizable to conventional radiography due to deficient ossification.

## Conclusion

PFFD is a rare congenital anomaly with a spectrum of affectation of the proximal two-thirds of the femur. The presence of two cases detected at early stages called for literature documentation. Besides, such early detection would have warranted timely intervention with consequent anticipated good childhood growth.

## Data Availability

Images used in this current study are available from the corresponding author on reasonable request.

## Abbreviations

CPFD: Congenital proximal femoral deficiency
CT: Computed tomography
GRE: Gradient echo
MR: Magnetic resonance
MRI: Magnetic resonance imaging
MRKH: Mayer–Rokitansky–Küster–Hauser syndrome
OMIM: Online Mendelian Inheritance in Man
PFFD: Proximal femoral focal deficiency
UUTH: University of Uyo Teaching Hospital
